# Context Matters: Team and Organizational Factors Associated with Reach of Evidence-Based Psychotherapies for PTSD in the Veterans Health Administration

**DOI:** 10.1007/s10488-017-0809-y

**Published:** 2017-06-09

**Authors:** Nina A. Sayer, Craig S. Rosen, Nancy C. Bernardy, Joan M. Cook, Robert J. Orazem, Kathleen M. Chard, David C. Mohr, Shannon M. Kehle-Forbes, Afsoon Eftekhari, Jill Crowley, Josef I. Ruzek, Brandy N. Smith, Paula P. Schnurr

**Affiliations:** 1 Center for Chronic Disease Outcomes Research, Minneapolis VA Health Care System, One Veterans Drive, Minneapolis, MN 55417 USA; 2 Department of Medicine, University of Minnesota, Minneapolis, MN USA; 3 Departments of Psychiatry, University of Minnesota, Minneapolis, MN USA; 4 Department of Psychology, University of Minnesota, Minneapolis, MN USA; 5 Dissemination & Training Division, National Center for PTSD, VA Palo Alto Health Care System, Menlo Park, CA USA; 6 Department of Psychiatry & Behavioral Sciences, Stanford University School of Medicine, Stanford, CA USA; 7 Executive Division, National Center for PTSD, White River Junction, VT USA; 8 Department of Psychiatry, Geisel School of Medicine at Dartmouth, Hanover, NH USA; 9 Evaluation Division, National Center for PTSD, VA Connecticut Health Care System, West Haven, CT USA; 10 Yale School of Medicine, NEPEC/182, 950 Campbell Avenue, West Haven, CT USA; 11 Cincinnati Department of Veterans Affairs (VA) Medical Center, Cincinnati, OH USA; 12 University of Cincinnati College of Medicine, Cincinnati, OH USA; 13 Center for Healthcare Organization and Implementation Research (CHOIR), VA Boston Healthcare System, Boston, MA USA; 14 Boston University School of Public Health, Boston, MA USA; 15 Palo Alto University, Palo Alto, CA USA

**Keywords:** Evidence-based practices, Mental health services, Posttraumatic stress disorder, Organization, Mixed methods, Implementation

## Abstract

Evidence-based psychotherapies for PTSD are often underused. The objective of this mixed-method study was to identify organizational and clinic factors that promote high levels of reach of evidence-based psychotherapies for PTSD 10 years into their dissemination throughout the Veterans Health Administration. We conducted 96 individual interviews with staff from ten outpatient PTSD teams at nine sites that differed in reach of evidence-based psychotherapies for PTSD. Major themes associated with reach included clinic mission, clinic leader and staff engagement, clinic operations, staff perceptions, and the practice environment. Strategies to improve reach of evidence-based psychotherapies should attend to organizational and team-level factors.

**Electronic supplementary material:**

The online version of this article (doi:10.1007/s10488-017-0809-y) contains supplementary material, which is available to authorized users.

## Introduction

Practice guidelines endorse specific trauma-focused psychotherapies as first-line treatments for Posttraumatic Stress Disorder (PTSD; Foa et al. 2009; Forbes et al. 2010; Department of Veterans Affairs (VA)/Department of Defense 2010), including prolonged exposure (PE; Foa et al. 2007), cognitive processing therapy (CPT; Resick et al. 2014), and Eye Movement Desensitization and Reprocessing (Shapiro 2001). These evidence-based psychotherapies generally produce greater improvements in PTSD symptoms than do either non-trauma-focused psychotherapies or medications (Lee et al. 2016). Yet, they are often underused (Becker et al. 2004; Shiner et al. 2013; van Minnen et al. 2010).

The VA, which provided treatment to almost 612,000 veterans diagnosed with PTSD in 2015, has devoted considerable resources to integrating CPT and PE into routine care. CPT and PE are time-intensive (8–12 weekly sessions), manualized treatments (Foa et al. 2007; Resick et al. 2014). To develop capacity to deliver these treatments, VA’s primary emphasis has been on clinician training. VA began nationwide competency-based training for CPT in 2006 and for PE in 2007. As of August, 2015, more than 6300 VA mental health clinicians had received training in PE and/or CPT (VA 2016). At the national policy level, VA mandates that all veterans with PTSD have access to CPT or PE. Resources to support local implementation include evidence-based psychotherapy coordinators at each medical center and a national PTSD mentoring program (Karlin et al. 2010).

Despite efforts to promote widespread use of CPT and PE, there remains substantial room for improving reach of these treatments to VA patients with PTSD. Intervention reach can be considered in terms of the percent and representativeness of individuals within a defined population who receive it. Reach is one metric of the public health impact of a health promotion initiative (Glasgow et al. 1999). Measurement of reach of specific types of psychotherapy in VA is challenging because administrative billing records for psychotherapy indicate only session length and format, not treatment approach. Using natural language processing of psychotherapy notes from six VA outpatient PTSD clinics in the Northeast, Shiner et al. (2013) found that only 6% of new patients diagnosed with PTSD received either CPT or PE during the first 6 months of treatment. In a related study, Watts et al. (2014) reported that reach varied by site from a low of 4% to a high of 14%.

To facilitate system-wide tracking of reach of evidence-based psychotherapies, VA released electronic psychotherapy progress note templates that allow for structured chart documentation and data extraction. Review of national data based on CPT and PE templates indicates that 3–4% of all patients with a PTSD diagnosis received either CPT or PE (VA 2017). This is likely an underestimate of use of CPT and PE as not all clinicians use templates for documentation. Nevertheless, even allowing for underrepresentation of CPT and PE in national data, available information indicates that these trauma-focused psychotherapies are not routinely used to treat VA patients with PTSD.

Research on barriers to widespread adoption by providers and reach to patients of trauma-focused psychotherapies both within and outside VA has focused primarily on provider- and patient-level barriers, such as lack of clinician training, clinician concerns about patient motivation and readiness (Cook et al. 2014; Hamblen et al. 2015; Zubkoff et al. 2015), clinician misperceptions that exposure to trauma-related memories is potentially harmful (Becker et al. 2004; van Minnen et al. 2010), and patient avoidance of trauma-related memories (Hundt et al. 2015). Although the implementation science literature has long noted that local setting factors impact adoption and sustainment of new practices (Aarons et al. 2011; Benjamin Wolk et al. 2016; Damschroder et al. 2009), very few studies have focused on organizational or team-level factors that might affect implementation and sustained use of evidence-based psychotherapies.

A recent literature review of 20 studies examining PE and CPT implementation in VA settings found that only three studies considered team-level variables (Rosen et al. 2016). One study involved interviews with heads of outpatient PTSD clinics and found wide variation in how clinics selected and prepared patients for CPT and PE (Hamblen et al. 2015). That study did not measure actual reach of CPT or PE and thus did not look at the relationship between these clinic factors and implementation success. Watts et al. (2014) found lower reach of CPT and PE in clinics with highly organized systems of care. The investigators did not determine which specific elements of the clinic infrastructure led to reduced implementation. A study focused on VA residential PTSD programs found that greater use of CPT and PE was associated with mandates and incentives to deliver these treatments, dedicated time and resources, and leaders who helped providers receive training and who provided time, credit, and resources for delivering those treatments (Cook et al. 2015a). Residential PTSD programs, however, treat only about 1% of all VA patients diagnosed with PTSD and it is unknown how these findings apply to outpatient treatment settings. An understanding of factors that lead to successful implementation of CPT and PE in outpatient PTSD clinics could set the stage for policy and quality improvement programs to increase the reach of these trauma-focused evidence-based psychotherapies.

The British National Health System Sustainability Model holds particular promise for identification of organizational and clinic-level factors resulting in successful integration of CPT and PE into routine care. The model was developed using various sources, including research with project leaders, directors, clinicians and global health experts (Doyle et al. 2013). It consists of ten categories, referred to as factors, that play a critical role in sustaining an innovation in healthcare settings: (a) benefits beyond helping patients; (b) credibility of the benefits; (c) adaptability of the improved process; (d) effectiveness of systems to monitor progress; (e) staff involvement and training to sustain the process; (f) staff attitudes toward sustaining the change; (g) senior leadership engagement; (h) clinical leadership engagement; (i) fit with organization’s strategic aims and culture; and (j) infrastructure for sustainability (Maher et al. 2010). The ten factors are conceptually grouped into three larger domains: The “process” (e.g., credibility and adaptability), “staff” (e.g., leadership and staff), and “organization” (e.g., infrastructure and fit with aims and culture). The Sustainability Model was designed for practical application; it is used to identify strengths and weaknesses in an implementation plan and predict sustainability of an improvement initiative within a specific organizational and workgroup context so that remedies can be made (Doyle et al. 2013; Maher et al. 2010).

In this study, the Sustainability Model was used to qualitatively explore determinants of sustained implementation of CPT and PE in VA outpatient PTSD teams. This allowed us to identify context-specific examples of the Sustainability Model factors as well as determinants that might not fit neatly within the Sustainability Model. Although developed for the British National Health System, the Sustainability Model has been used to examine implementation and sustainability of VA’s Mental Health System Redesign initiative (Ford et al. 2011), Primary Care Mental Health Integration projects (Ford et al. 2012), and TBI/polytrauma system of care Family Care Map (Ford et al. 2014). We focused on implementation of CPT and PE in specialized outpatient PTSD teams, known as PTSD Clinical Teams, because VA mental health clinicians work in teams and PTSD Clinical Teams play a leading role in the delivery of specialized, disorder-specific care for PTSD (Karlin et al. 2010). Our primary objective was to identify specific organizational and clinic factors that promote high levels of reach of CPT and PE 10 years into VA’s CPT and PE dissemination and training initiatives. Although quantitative data was used to select sites and obtain contextual information about the teams under investigation, the primary method of data collection was semi-structured individual interviews. Qualitative methods are particularly well-suited for circumstances such as this in which extant research is limited and variables of interest involve complex, dynamic processes (Miles et al. 2014; Palinkas et al. 2011).

## Methods

### Study Design and Sample

The VA Central IRB approved all aspects of this research. This study used Rapid Assessment Process methodology that is designed to obtain in-depth, qualitative information in a relatively brief amount of time (Beebe 2001, 2014). Because adjustment to field conditions is integral to this method, there is not one prescribed way to conduct a Rapid Assessment Process. Rather, a Rapid Assessment Process is defined by the following techniques: (a) close collaboration with a field liaison, (b) triangulation, and (c) iterative data collection and analysis. The field liaison is a member of the group being studied who helps the research team understand the site and provides logistic support prior to and during each site visit. Triangulation is achieved in various ways. First, there is triangulation by researchers who serve as the instruments in qualitative research; having multiple researchers’ interview and either write or review summary interview notes allows the collection and examination of the data by several individuals. Second, triangulation is achieved by having different data sources. Triangulation by researchers and data source aims to identify converging themes and achieve thematic saturation by content area. The iterative process of data collection and analysis means that the researchers analyze data after each interview and then use the subsequent interviews to fill in gaps, explore emerging content areas, and verify or disconfirm themes. Rapid Assessment Process also includes review of quantitative data, such as reports, records, or statistics.

As is standard in qualitative research, sampling was done by the outcome of interest (Mahoney and Goetz 2004). Specifically, to discover factors that facilitated and impeded sustainability, PTSD teams were selected to reflect a range of geographic regions, patient volume and reach of CPT and PE. Because we were particularly interested in identifying factors associated with high levels of adoption and sustained use of CPT and PE, we selected a larger number of high than medium and low reach teams.

VA administrative data was used to identify our recruitment pool for purposive sampling. First, we identified all outpatient PTSD teams by location and PTSD patient volume. Second, to characterize teams in terms of CPT and PE reach, we examined the content of psychotherapy chart notes. Because manual chart review is impractical for application to a large number of notes, we used an automated procedure that involved regular expression searches for CPT- and PE-related terms (including the names of the therapies themselves). We applied these regular expression searches to group and individual psychotherapy notes for all therapy patients with PTSD seen on an outpatient PTSD team over an 18-month period (*N* = 110,491) and thereby determined whether each patient had received at least one CPT- or PE-related session. Regular expression searches likely resulted in an over-estimate of reach as we could not exclude sessions in which CPT and PE were discussed but not delivered. However, this method provided sufficient information for us to group teams into relatively high, medium, and low reach categories for sampling. The high reach group included teams with reach exceeding 33% (*n* = 22), the medium reach group included those with reach between 20 and 33% (*n* = 38) and the low reach group included teams with estimated reach below the national mean of 20% (*n* = 80). We recruited teams from the high, medium, and low reach groups in different regions until we obtained nine sites that demonstrated the desired diversity. During pre-site visit calls with local field liaisons, we learned that one site had two separate outpatient PTSD teams. Thus, our final sample included ten teams from nine medical centers across the U.S.

We used natural language processing to more accurately characterize reach of CPT and PE in the ten selected teams near the time of interview data collection. In particular, building on the work of Shiner et al. (2013), we created our own specialized natural language processing pipeline that had high performance as measured by precision, recall, and F-measure for both CPT and PE when tested against a set of manually annotated notes (natural language processing system performance metrics available upon request). We applied our natural language processing system to individual and group psychotherapy notes for patients with PTSD seen on the selected PTSD teams over the 12-month period during which most site visit interviews took place (October 1, 2014 through September 30, 2015).

### Data Collection

Approximately 2 months before interviews, we conducted anonymous staff surveys of clinicians on the PTSD teams to assess CPT and PE training history, team processes and CPT and PE sustainability using standardized measures. The surveys gave the interviewers preliminary information about the team which they could then triangulate with interview data. Findings based on quantitative analysis of the surveys, however, will be described in separate manuscripts.

The project coordinator invited the identified staff to participate in individual interviews using email and telephone. Almost all interviews were conducted by two of our four member interview team and always included at least one clinical psychologist with expertise in PTSD care, qualitative research, and implementation science (either NAS or CR). Informed consent was obtained prior to the interviews. In preparation for the staff interviews, we interviewed three individuals with roles in overseeing the CPT and PE rollouts nationally and one VA staff who led an initiative to spread evidence-based psychotherapies in a site not included in our sample. For our site visits, we interviewed between 7 and 15 VA staff from each of the nine selected medical centers between October 2014 and December 2015. For seven out of the nine medical centers, most interviews occurred face to face during 2–3 day site visits. Due to problems scheduling visits at two sites (one low and one medium reach), interviews occurred over the telephone. We also interviewed three staff with leadership responsibility for mental health care at the regional level. These interviews took place over the telephone. Thus, in total, we interviewed 93 medical center staff and three regional mental health leads (*N* = 96). Data Supplement 1 presents professions of those interviewed by site. All but six interviews were audio-recorded; these six participants did not consent to audio-recording. The interview team took detailed notes during each interview.

The informed consent specified that the purpose of this research study was to identify factors that interfere with and promote adoption and sustainability of CPT and PE. Interviews were conducted using a semi-structured interview guide that included probes for the ten factors included in the Sustainability Model (Doyle et al. 2013; Maher et al. 2010). Participants were also invited to discuss barriers and facilitators of CPT and PE implementation and sustainability not included in the interview guide.

The interviewers reviewed VA policy documents related to the evidence-based psychotherapies training initiatives. In addition, six sites provided the interviewers with examples of patient brochures or slides used to describe their PTSD teams, manuals for groups conducted to educate patients about PTSD and clinical services available, or copies of scientific articles the PTSD team members had published based on data collected as part of clinical care. As is typical using Rapid Assessment Process methodology (Beebe 2001, 2014), the interviewers verbally presented emerging findings based on preliminary site summaries (see Data Analysis) to the PTSD team and requested feedback at the end of six of the nine site visits. This allowed the interviewers to check facts, fill in missing information and verify emerging conclusions. During these feedback sessions, the interview team also made and later recorded observations that further explicated the themes identified. Clinicians affiliated with the teams but not interviewed participated in feedback sessions.

### Data Analysis

Analysis involved the following iterative steps: (a) after each interview, the interviewers jointly created a post-interview log that detailed the findings per participant in each area of inquiry (e.g., per each Sustainability Model factor), new content areas for further inquiry, and observations. Thus, coding based initially on the Sustainability Model began at the time of creating the first post-interview log. (b) After the last interview at a site, the interviewers prepared a consensus document summarizing themes across the interviews to create a preliminary site summary. In addition, the interviewers identified key sections for transcription to extract representative quotes. (c) Within the week following the site visit, the lead interviewers (NAS and CR) reviewed the preliminary site summary, interview audio-recordings, and documents the site provided (e.g., program brochures) to refine and expand the site summaries. The other member of the interview team reviewed, verified and elaborated the site summary. At each step, we grouped the information into the Sustainability Model factors whenever possible and noted additional themes as well as the connections among themes.

We used our consensus-based site summaries to perform inter-case analyses. Specifically, we used constant comparison (Glaser and Strauss 1967) to compare and contrast high, medium, and low reach sites in terms of themes and looked for exceptions to any patterns identified. Although interview questions were based on the Sustainability Model, emergent themes were identified to reflect participants’ experiences. We grouped findings into major themes using terms that reflected participants’ own language and experiences rather than the Sustainability Model’s larger domains of “process” “staff” and “organization” so that the results and interrelationship among major themes would be readily understood by stakeholders (e.g., decision makers and clinicians). This too is consistent with Rapid Assessment Process methodology (Beebe 2001, 2014).

## Results

Site and PTSD team characteristics, including reach, are presented in Table 1. We studied five high (two at the same medical center), two medium, and three low reach PTSD teams situated in sites that varied geographically and in terms of the number of patients with PTSD. As shown in Table 2, factors associated with reach of CPT and PE were grouped into five major themes, four of which had a set of dimensions that characterize their meaning. The last column of Table 2 shows the Sustainability Model factor that corresponds to each dimension. All but two dimensions (team mission and fit with culture outside the organization) corresponded to Sustainability Model factors. One Sustainability Model factor (infrastructure for sustainment) corresponds to five dimensions that span two major themes, demonstrating the complexity of this construct in this context.

**Table 1:** Study site and PTSD team characteristics

Site and team labels^a^	US census region	Number PTSD patients seen in medical center	Number PTSD patients seen by PTSD team^c^	Number PTSD patients who received psychotherapy on PTSD team	Number providers on PTSD team	Reach on PTSD team^d^ (%)	Reach category
7	Midwest	3119	1,083	705	22	58.9	High
5a	West	5803	729	702	5	38.6	High
5b			231	216	3	56.5	High
2	Midwest	2027	389	372	10	55.9	High
4	South	6880	2293	1533	35	42.0	High
3	West	1762	512	400	8	31.7	Medium
9^b^	Midwest	1441	373	184	4	28.8	Medium
6	Northeast	5042	1470	690	19	17.7	Low
1	Northeast	2370	1264	621	16	15.3	Low
8	South	2986	1570	838	18	14.0	Low

*PTSD* posttraumatic stress disorder. All numeric values based on administrative data from fiscal year 2015

^a^Site numbers reflect the order in which site visit interviews were conducted

^b^Medical center designated rural; all others designated urban

^c^Number PTSD patients seen by PTSD team is a subset of number PTSD patients seen in facility

^d^Reach was calculated as the number psychotherapy patients with PTSD seen on the PTSD team who received either CPT or PE divided by the total number of psychotherapy patients seen on the PTSD team

**Table 2:** Major themes and dimensions associated with reach of evidence-based psychotherapies for PTSD

Major themes	Concepts	Dimensions	Corresponding sustainability model factor
Clinic mission	Goals or purpose of the team		None
Team engagement	Demonstrated commitment to team mission		
		Clinic leader engagement	Clinic leader engagement
		Staff involvement and training	Staff involvement and training
		Staff attitudes toward sustainment^a^	Staff attitudes toward sustainment
Clinic operations	Clinic policy and procedures to support and enact the team mission		
		Patient selection	Infrastructure for sustainment
		Patient preparation	Infrastructure for sustainment
		Effectiveness of system to monitor progress	Effectiveness of system to monitor progress
		Peer consultation	Infrastructure for sustainment
		Scheduling flexibility^a^	Infrastructure for sustainment
Perceptions of evidence based psychotherapies for PTSD	Beliefs about CPT ad PE in terms of benefits and adaptability		
		Credibility of clinical benefits	Credibility of benefits
			Benefits beyond helping patients
		Adaptability of CPT and PE^a^	Adaptability of the improved process
The practice environment	Context outside the clinic		
		Infrastructure outside the clinic	Infrastructure for sustainment
		Senior leadership engagement	Senior leader engagement
		Fit with the organization’s strategic aims and culture^a^	Fit with the organization’s strategic aims and culture
		Fit with culture outside of the organization^a^	None

*PTSD* posttraumatic stress disorder

^a^All teams shared similar views of this dimension

Below we compare and contrast high and low reach teams in terms of major themes and dimensions. Medium reach teams were grouped with high or low reach teams, depending on which group they were most similar to on a given dimension. Medium reach teams are grouped with high reach teams for major themes 1, 2 and 5; they are grouped with both high and low reach teams for major themes 3 and 4. We also describe dimensions shared across all teams.

### Theme 1: Clinic Mission

**Fig. 1:**
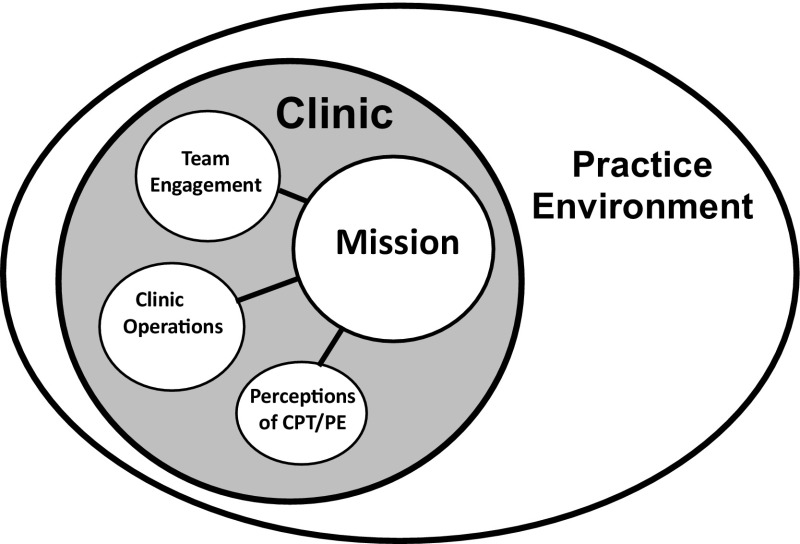
Centrality of clinic mission

The most critical difference between clinics with high and low reach of CPT and PE was the team’s definition of clinic mission. Mission is a term used in the military to describe a task including its purpose and associated actions. The centrality of clinic mission to the other major themes is depicted in Fig. 1.

#### High and Medium Reach Teams

All high reach teams identified as evidence-based psychotherapy clinics with the primary mission being to deliver PE and CPT in weekly therapy sessions during a treatment episode. This coherent sense of purpose was seen even in high reach teams that offered other structured short-term psychotherapies in addition to CPT and PE. Medium reach teams had adopted or partially adopted the evidence-based psychotherapies mission more recently (within the past 1–2 years) in order to facilitate patient access to CPT and PE. One medium reach team described embracing a model of care that included CPT and PE as well symptom management groups. In high and medium reach teams, patients were followed by therapists for 6 months to 2 years and were then discharged to either no treatment or to a lower level of care. The quote below illustrates the centrality of the evidence-based psychotherapies mission for high reach teams:

It’s always been made clear the kind of program this is, we are evidence-based, we are cognitive-behavioral based. If that does not fit with your orientation, that’s OK but we’re not going to change, that’s what you are going to be doing if you come in here. So it’s just aligned very nicely with the theoretical orientation of the program and the people (clinical staff) who come on board.

#### Low Reach Teams

The team mission and treatment model were more varied within low reach teams. Two of the low reach teams described themselves as general mental health clinics for veterans with PTSD or a subset of patients with PTSD. Another low reach team described itself as specialty mental health for military-related PTSD. All low reach teams offered CPT and PE and other mental health treatment options, including symptom management skills groups as well as long-term and supportive therapy approaches. Some offered complementary therapies, such as Art Therapy. Patients were followed by the team for the long-term and discharge from the clinic was rare. The quote below illustrates the clinic vision in a low reach team:

There are (PTSD Clinical Teams) where people only come for the evidence based treatment and they get returned to their mental health clinic provider or primary care provider…Our service has never been set up this way…The shared value that we attempt to promote is that we are treating people and not symptoms, that we have the veteran until they do not need us anymore…So, how do we know when they are done? That’s a really important question.

### Theme 2: Team Engagement

#### High and Medium Reach Teams

##### Clinic Leader Engagement

All high and medium reach teams reported that their team leader was highly engaged in sustained implementation of CPT and PE. The team leader had clinical expertise in CPT and/or PE and was described as a champion. One leader of a medium reach team was a strong proponent of CPT and PE yet also endorsed approaches that focus on symptom management. In all high reach teams, the team leader played a key role in establishing the team mission and operations. Teams in the midst of leadership transitions saw the loss of strong clinic leaders as a threat to sustainment of their mission and associated infrastructure.

##### Staff Involvement and Training

Generally, the clinic mission came from the team leader or site’s chief of mental health. In only one medium reach site were the clinicians on the team involved in the decision to prioritize delivery of CPT and PE. Yet, staff in all high and medium reach teams reported commitment to time-limited evidence-based psychotherapies. Even teams that included psychiatrists as well psychotherapists consistently described pharmacotherapy as secondary to CPT and PE. Most if not all psychotherapists were trained in CPT and PE and new staff were required to know or learn them. As this mental health chief explained:

We have enthusiasm and we engender that in the staff that we hire…I wouldn’t think about hiring someone for the (PTSD Clinical Team) clinic that wasn’t willing to devote a lot of time to PE and CPT. I think it’s just ingrained pretty well now.

#### Low Reach Teams

##### Clinic Leader Engagement

Leaders of the low reach teams had not been trained in CPT or PE and were not as strong champions for these evidence-based psychotherapies. Although they saw them as useful treatment options, they did not view them as fundamentally better than other treatments. They described themselves as having administrative concerns other than or in addition to ensuring that patients with PTSD receive an evidence-based psychotherapy, such as timely access and long-term mental health care.

##### Staff Involvement and Training

In low reach clinics, there was more variability in staff commitment to evidence-based psychotherapy although some to most psychotherapists were trained to deliver CPT and/or PE and saw CPT and PE as valuable treatment options for some patients. Some psychotherapists trained in CPT and/or PE were frustrated by their leader’s lack of commitment to evidence-based psychotherapy. In general, psychiatrists played a more predominant role in low reach teams.

#### All Teams

##### Staff Attitudes Toward Sustainment

In general, staff on all teams believed that CPT and PE would continue to be a treatment option for patients with PTSD because VA policy requires their availability. At the same time, staff noted the importance of VA’s continued commitment to training to ensure sustainability.

### Theme 3: Clinic Operations

#### High and Medium Reach Teams

##### Patient Selection

All high reach teams and one medium reach team had developed screening procedures to help ensure that the patients who came to their clinic for an intake were interested in the psychotherapies they had to offer before scheduling an intake session. The screening procedures included specifying patient interest in a trauma-focused treatment as a referral criterion and/or requiring patients to undergo an orientation session in which clinic services, including CPT and PE, were described. Thus, patients entering these clinics were likely to be receptive to the short-term evidence-based psychotherapies that the clinic offered:

Once they (patients with PTSD) are sent to us, they go through an orientation group, so they are well versed in what we do here and what we have to offer so they have the choice of, “Is this something you want to engage in?”, or, “Here’s your menu of options.”…An informed patient is an activated patient.

##### Patient Preparation

Some high reach teams offered CPT or PE immediately after an intake. Other high reach teams provided a four-session psychoeducational group prior to beginning CPT or PE. One medium reach site used to offer a 10-week psychoeducational group before CPT or PE, but had transferred this group to their general mental health clinic where patients generally completed it before referral to the PTSD team. That team, however, reported difficulty getting patients to agree to begin CPT or PE right away, so most patients completed one or more symptom management group before engaging in CPT or PE.

##### Effectiveness of System to Monitor Progress

Most high reach teams designed and implemented systems and clinic databases to monitor both treatment process and patient outcomes. Managers of most high reach teams described using outcome data to demonstrate the value of CPT and PE to medical center leadership to maintain support for the resources dedicated to their clinic, as illustrated with the quote below:

We have a specific database…where we track all of our outcomes, pre, mid, and post treatment, assessments, self-report. We also measure drop out, non-engagement, referrals to other programming so that we can really be accountable to our outcomes, we can actually show people what we do and feel comfortable with that and I think that really fosters that evidence-based quality of our services.

##### Peer Consultation Group

All high reach and one medium reach site reported having CPT and PE peer consultation groups in which clinical issues were discussed. Peer consultation groups were distinct from administrative meetings. Staff on one medium reach team emphasized that staff had protected time to attend weekly peer consultation group. These groups were seen as a valuable resource dedicated to supporting high quality implementation.

#### Low and Medium Reach Teams

##### Patient Selection

All low reach teams and one medium reach team did not have procedures for selecting patients based on their interest and willingness to do trauma-focused or other evidenced-based psychotherapies. One low reach team had a script that informed patients that, “Treatment in this clinic involves in-person visits with a psychiatrist as well as a therapist and typically involves more frequent appointments.” This script was developed to encourage patients who might be receptive to CPT and PE to select the clinic for their mental health care.

##### Patient Preparation

All low reach teams had patients complete a psychoeducational group before they began CPT or PE. Some offered four-session psychoeducation groups for this purpose. One offered an orientation session before and after the intake as well as a 10-week psychoeducational group program before offering CPT or PE. The rationale for having patients complete educational groups before offering them CPT or PE was to build interest and develop skills to facilitate successful engagement in these evidence-based psychotherapies.

##### Effectiveness of System to Monitor Progress

While some low and medium reach teams monitored some processes of care (e.g., number of patients receiving CPT or PE), they did not monitor clinical outcomes. Outcome monitoring occurred at only one low reach site and was restricted to monitoring PE and CPT outcomes. This effort was led by individual clinicians and was not part of a clinic-wide system. However, staff at low reach sites recognized the importance of systems to monitor progress, as demonstrated by this quote from a team Medical Director:

One thing that we do look at is that the number of people going into evidence-based psychotherapies is much smaller than the number of people coming into our clinic. Where do they go? I don’t know where they go. What are we doing with them? I don’t know. How many of them come a few times and stop? I don’t know. If we could ask any of those questions in again a relatively real-time, contemporary way, we could get a picture of what is going on, we would be in a better position to look at our processes.

##### Peer Consultation Group

Only one low reach site had a peer consultation group to support high quality delivery of CPT and PE. This had been recently introduced as part of a focused effort to increase provider adoption of all evidence-based psychotherapies that VA has disseminated.

#### All Teams

##### Scheduling Flexibility

Nearly all teams, regardless of their reach, noted that therapists need to be able to manage their own schedules to effectively deliver evidence-based psychotherapy. This was seen as particularly important for PE, which requires 90-min individual sessions (Foa et al. 2007). Staff in some teams had control over their own schedules. Yet, some low and high reach teams reported that the inflexibility in the scheduling system in their facilities made it difficult to schedule psychotherapy sessions for the duration and at the frequency required to deliver evidence-based psychotherapy. Changes in scheduling procedures were made at one low reach site as part of an effort to increase reach. As one clinician explained:

That was one of the big changes…and probably the most important one was…psychologists or providers they could schedule as they wanted…That was probably the most important thing in doing more evidence-based therapy.

### Theme 4: Perceptions of CPT and PE

#### High and Medium Reach Teams

##### Credibility of Clinical Benefits

Staff in nearly all of the high reach teams described CPT and PE as being beneficial for the majority of patients with PTSD. Moreover, staff saw these treatments as more effective than other psychotherapies. Staff at one high reach team noted that while benefits were strong, they may not be permanent and that a significant proportion of patients will need to resume mental health care at a later time point after completing CPT or PE.

As one clinician noted, “They [CPT and PE] don’t work for everybody, but they work more times than not. And with the volume of patients that we have, trying to deliver something that works for the majority, um, is a good idea, just makes sense.” Staff in most high reach sites believed that a high proportion of patients (at least those screened into their clinic) were interested in CPT or PE. However, high reach teams varied in the extent to which they reported difficulties retaining patients in these treatments.

##### Benefits Beyond Helping Patients

Staff in high and medium reach teams believed that use of CPT and PE benefitted the clinic as well as patients. Staff noted that provision of evidence-based psychotherapies helps with staff recruitment because trainees from different disciplines want to learn to deliver them. CPT and PE were described as increasing morale and reducing burnout because clinicians see their patients improve. As this clinician explained, “I’m not sure that I could’ve continued to do my job for as long as I have if we weren’t doing a lot of evidence-based psychotherapy…I see people improving.” Staff in high and medium reach sites also reported that the time-limited nature of CPT and PE meant that the clinic could discharge patients or see them much less frequently and thus make room for new patients.

#### Low and Medium Reach Teams

##### Credibility of Clinical Benefits

Staff at all low reach sites and one medium reach site reported that although CPT and PE were credible, they did not live up to expectations. Staff in low reach sites described the clinical benefits as “partial” or “not universal.” As one clinician explained, “They’re overselling the treatments, oversimplifying what’s needed. They’re great treatments, they’ve generated a lot of research and pubs, they can be manualized, but they’re just a small piece of a very large puzzle.” Staff at one low reach site believed that the clinical benefits of CPT and PE were credible to those delivering these psychotherapies, but less clear to those who do not deliver them. Staff in low and medium reach sites also expressed the belief that CPT and PE alone are not sufficient, and that many patients with PTSD need or want mental health services other than, in addition to, and after completion of CPT or PE.

Among patients who start CPT or PE, drop out was reported to be a large problem. As this clinician expressed:

We all say these are the Cadillacs, these are the best treatments, we really try our best to sell them but it’s still not a high number of people that are jumping up to participate in them and when they do participate, then we have, it feels like…there’s a high number of drop outs.

Staff at some low and medium reach sites believed that patients became interested in CPT and PE only after forming solid therapeutic relationships and/or developing distress tolerance skills. Therefore, it was believed that while CPT and PE are a valuable treatment option, other treatments should not be devalued.

##### Benefits Beyond Helping Patients

There was variation across staff in low reach sites with regard to the perceived benefits for clinic performance. Some staff shared the positive views expressed in high reach sites, whereas others within the same team did not see strong benefits to staff or the clinic. This latter group stated that CPT and PE increased provider burden because of the time required to prepare for sessions, sessions being more emotionally draining, and, in the case of PE, difficulty fitting the standard 90-min sessions into their schedules. Overall, in low reach sites, evidence-based psychotherapies were superimposed on other general mental healthcare responsibilities, such as crisis or case management and care coordination for complex patients with multiple co-morbidities. Some staff in low and medium reach teams valued the ability to select treatment approaches for their patients and would not want to be part of clinic that only offered CPT and PE. Staff at one low reach site worried that initiatives to expand use of CPT and PE would undermine clinician autonomy and ultimately patient care. Moreover, because staff in low reach sites viewed PTSD as a chronic condition requiring continuing care after completion of CPT and PE for most patients, provision of these therapies was not seen as helping the clinic improve its efficiency. This point of view was echoed by a clinician from a medium reach site:

Sometimes the selling point of efficiency and getting patients off of caseloads and so on and so forth, is really probably oversold in my experience…I certainly think the treatments are effective and patients benefit from them, but I think those cases often require some continued long-term follow-up.

#### All Teams

##### Adaptability of CPT and PE

Generally, clinicians described trying to follow the CPT and PE manuals, with some flexing to address patient-specific needs. One high reach team reported significant changes in delivery format; staff in one low reach team described some clinicians implementing only portions of the treatment protocols. Concern about treatment fidelity varied across teams, with some being more focused on fidelity than others. Some staff believed that more research is needed to determine how to adapt the treatments for different types of patients; others believed that lack of adaptability contributed to drop out. Staff at one high reach site emphasized that although the treatments were not adaptable, clinic processes were and thus should be adjusted to make it easier to deliver evidence-based psychotherapies.

### Theme 5: The Practice Environment

#### High and Medium Reach Teams

##### Infrastructure Outside the Clinic

Staff working within high and medium reach clinics described being able to refer their patients to other mental health teams, primary care and other programs within the medical center and seemed much less burdened by addressing patients’ psychosocial problems in addition to delivering psychotherapy or pharmacotherapy. Staff members with one medium reach team were able to shift responsibility for a PTSD psychoeducational group to another team to free up time for delivering CPT and PE.

##### Senior Leadership Engagement

Generally, senior hospital leadership was seen as somewhat removed from mental health care. However, at most high and medium reach sites, the chief of mental health was supportive of the team’s mission to prioritize CPT and PE. For one medium reach site, the chief of mental health was responsible for initiating the transformation of the PTSD team from a general mental health clinic that provided long-term care to patients with PTSD to an evidence-based psychotherapies for PTSD clinic. A clinician explained:

Our mental health care line manager was pretty gung-ho and excited about evidence-based psychotherapies from the get go. And all I have to do is to say we need to do this to better implement it and he says, “OK do it”…He was very supportive…of our being able to block weekly slots.

The other medium reach team reported that although clinic staff initiated the change in mission, the team leader obtained the support of the chief of mental health before implementation. Lack of support from the chief of mental health was considered a significant problem for high reach sites experiencing team leadership transitions.

#### Low Reach Teams

##### Infrastructure Outside the Clinic

Most low reach teams reported limited availability of mental health or psychosocial services for PTSD patients outside of their clinics. Rather, they described themselves as providing all types of mental health and psychosocial care for patients with PTSD for as long as they needed and wanted it. Clinicians on these teams reported performing case management responsibilities (whether or not they were formally designated as case managers) and spending considerable time identifying resources for their patients when they had psychosocial crises. Competing task demands limited their ability to focus on evidence-based psychotherapies. This clinician explained the challenges clinicians face when the PTSD team is expected to provide all types of services for their patients:

We also, once somebody’s assigned to our clinic, they’re always assigned to our clinic. It’s really difficult to say let us just treat the PTSD and then-, we don’t have a general mental health (clinic)…and that (not having a general mental health clinic) is a *massive* barrier…Somebody might be with me for years and years and I’ve done CPT with them but they’re still sort of mine and if they’re in crisis they come back to me.

##### Senior Leadership Engagement

At most low reach sites, the chiefs of mental health did not see the value or feasibility of implementing a PTSD team that focused primarily on CPT and PE delivery either because of concerns about the limited efficacy of CPT or PE for the majority of patients with PTSD or because of the view that the medical center did not have enough staff to create a specialized team to only treat PTSD patients appropriate for and interested in CPT and/or PE. At one low reach site, however, the chief of mental health had recently established structures to promote use of evidence-based psychotherapies for PTSD and other conditions across all mental health teams within the medical center.

#### All Teams

##### Fit with the Organization’s Strategic Aims and Culture

Staff had mixed views of how CPT and PE fit within the culture in the medical centers in which they worked. In general, CPT and PE were seen as fitting the medical centers and the affiliate university’s focus on evidence-based care. At the same time, staff at most low reach sites and one high reach site believed evidence-based psychotherapies did not fit with the emphasis on psychodynamic psychotherapies within the larger community. Generally, with the exception of one high reach site, evidence-based psychotherapies were not a major part of the broader practice environment. Staff from some high reach sites described how the team’s episode of care model appeared to be spreading to other mental health teams and how the PTSD team’s approach to measuring process and outcomes was setting the standard for measurement-based care for mental health in the medical center.

##### Fit with Culture Outside of the Organization

Some staff described how evidence-based psychotherapy represented a culture change. They noted that VA patients with long histories of receiving open-ended supportive psychotherapy and their therapists may not find an episode-of-care model or the possibility of recovery credible. Others believed that the media has promoted the perception that PTSD is chronic and not treatable. The VA disability program was seen as reinforcing a view of PTSD that conflicts with a recovery orientation. Some staff believed that it is easier to change expectations regarding treatment among the newer veterans and newer staff who do not believe that PTSD and mental health treatment need to be life-long. As one clinician explained:

There are a lot of people who have been here a long time, who do better with the evidence-based treatment but still expect to come to the VA on a weekly basis cause this is their, this is what they’ve been doing for the last 25 years. I think we are able to make changes with the people who are here for the first time, their expectations are different.

On the other hand, staff noted that veterans’ views of PTSD and PTSD treatment can change and that when a patient benefits from CPT or PE, he or she is a more convincing source of information about the effectiveness of evidence-based psychotherpy than clinicians or promotional material.

## Discussion

The most striking difference between VA outpatient PTSD teams with high and low reach of CPT and PE was that all high reach teams were organized around a primary mission of delivering time-limited evidence-based psychotherapies for PTSD. The emergence of this theme was noteworthy because it was not one of the factors included in the Sustainability Model that informed the semi-structured interview guides used here. Rather, team mission emerged organically. Team mission is superordinate to the Sustainability Model’s factor “infrastructure for sustainment” in that it guides decisions regarding elements of infrastructure internal to the team (e.g., work processes). Team mission is also distinct from the Sustainability Model construct of “fit with organization’s strategic aims and culture.” Fit with aims and culture suggests an innovation being compatible with the existing ecosystem, like a non-native seed growing well in a new environment. Team mission is more suggestive of a purpose-built mini-ecosystem to support the innovation, like having flooded fields specifically designed for growing rice within a larger landscape. Thus rather than being subsumed under these factors, team mission functioned as a fundamental organizing principle for both clinic infrastructure and team culture. Indeed, clinics that organized around the evidence-based psychotherapies for PTSD mission adapted their work group to these interventions. A large body of research has discussed how tailoring innovations to the local organization can improve implementation (Damschroder et al. 2009; Greenhalgh et al. 2004; Rogers 1995) and sustainability (Chambers et al. 2013; Maher et al. 2010; Scheirer and Dearing 2011). Other studies have examined how system redesign can improve clinical outcomes (Reid et al. 2013); fewer studies have considered how embracing a clinical innovation can provide an impetus for alteration in the organization of the work group (Kirsh et al. 2008).

Several factors may explain why organizing the clinic in support of a defined team mission emerged as important in this particular context. First, psychotherapies like CPT and PE are resource-intensive, complex interventions; they involve greater behavioral changes than implementing a new screening tool or substituting one medication for another. Second, we found that CPT and PE were not viewed as very adaptable. With a few exceptions, staff described attempts to maintain fidelity to these interventions as manualized. The necessity to change how the team operated may be heightened when innovation-organization fit cannot be improved by tailoring the intervention. Third, PTSD clinics were established as disorder-specific specialty clinics before the emergence of VA’s CPT and PE training initiatives. Thus, the evidence-based psychotherapy mission involved further specialization around this diagnosis. Although the importance of team mission may be specific to specialized outpatient PTSD teams, other researchers have examined the linkage between organizational social context, which includes organizational culture and climate, and delivery of evidence-based care (Aarons et al. 2012; Glisson et al. 2008). Future research should determine whether team mission is equally important to implementation and sustainability of evidence-based care in other highly specialized practice settings or whether team mission plays a role in implementation and sustainability of innovations in practice settings that address a diverse set of medical problems.

Clinic and medical center mental health leadership played an instrumental role in championing and implementing the mission of high reach teams. This finding is consistent with the Sustainability Model (Maher et al. 2010). Other research indicates that leadership characteristics associated with implementation and sustainment include being knowledgeable about evidence-based interventions, supportive of staff in the implementation process, proactive in problem-solving implementation issues and steadfast through the challenges of implementation (Aarons et al. 2016; Damschroder et al. 2009; Weiner 2009). Staff in high reach teams described clinic leaders as promoting a strong implementation climate (Damschroder et al. 2009) by communicating that use of CPT and PE was expected and would be supported and rewarded. Future implementation efforts might be expanded to include training for clinic and medical center mental health leaders on methods to promote implementation readiness and a supportive implementation climate for evidence-based psychotherapy.

Staff beliefs about the benefits of CPT and PE were more positive in high versus low reach teams. However, our findings indicate that the credibility of benefits construct is complex. Specifically, although most staff members believed that CPT and PE are potentially effective and should be offered, there was variation across teams and in some cases within teams regarding beliefs about the number of patients with PTSD who would be interested and benefit clinically from these treatments. Staff at low reach teams believed that the treatments have been oversold as being suitable and effective for more patients than they are, and that other kinds of treatment were necessary to address, comprehensively, the needs of veterans with PTSD. In this way, staff at low reach teams saw the potential population impact of CPT and PE as relatively low. This point of view resembles that of some experts (Steenkamp et al. 2015). Other studies have reported that low patient “readiness” for trauma focused psychotherapy limits use of CPT and PE in VA (Rosen et al. 2016; Zubkoff et al. 2015). However, patient interest in CPT and PE may depend in part on how patients are educated about treatment options. For example, two studies demonstrated that use of decision aids can increase veterans’ likelihood of initiating CPT and PE (Mott et al. 2014; Watts et al. 2015). Regardless, findings from this study indicate that measures of intervention characteristics (e.g., Cook et al. 2015b) should include perceptions of patients’ receptivity to the intervention.

The infrastructure for sustainability domain of the Sustainability Model is quite broad, including staff, job descriptions, policies and procedures. Participants in the present study differentiated two levels of infrastructure. One was policies and procedures within the clinic which we termed “clinic operations.” High reach teams had implemented clinic operations to facilitate delivery and sustainability of CPT and PE. Low reach teams, in general, had added services to prepare patients for CPT and PE but largely grafted CPT and PE onto clinic processes that did not fit the short-term evidence-based psychotherapy model. The second was infrastructure in the organization (outside the team) for patients with mental health disorders. Specifically, PTSD teams could only adopt an evidence-based psychotherapy mission in medical centers where other teams provided supportive services or ongoing care that patients might need. This finding suggests that senior leadership should review the design of mental health services as a whole if they are interested in sustaining high levels of reach of evidence-based psychotherapies within specific mental health teams. We recommend that the Sustainability Model be adapted to include assessment of infrastructure for sustainment in both the inner and outer setting contexts.

Although enactment of the team mission required cooperation with other programs and support from mental health leadership in the medical center, it did not depend on a site-level culture of evidence-based care. Only one of the high reach teams was embedded in a practice environment where the culture outside the team fully embraced evidence-based mental health care as the norm. Staff on high reach teams also talked about how the evidence-based psychotherapy model was spreading to other mental health teams, suggesting that innovation success in one setting can influence other settings (Dückers et al. 2014). Furthermore, enactment of team mission did not require a culture outside the organization to support evidence-based psychotherapies for PTSD. In fact, staff across teams of varying levels of reach believed that the broader culture hinders widespread acceptance of CPT and PE as frontline treatments for PTSD.

High and low reach teams differed in their use of monitoring systems, a dimension which we considered part of the clinic’s infrastructure. Measurement of outcomes over time has been shown to support sustainment (Glasgow et al. 2012). In the absence of routine outcome data, it may be challenging to convince clinic and senior leaders that it is important to restructure a low reach PTSD team to fit an evidence-based psychotherapy mission treatment model. In the absence of outcome data, it may also be difficult to convince leaders to continue to support a high reach team’s evidence-based psychotherapy mission and clinic infrastructure when faced with competing demands. Outcome monitoring could also address staff concerns about the credibility of CPT and PE benefits and facilitate identification of promising context-specific adaptations (Chambers et al. 2013). Many teams had developed unique databases, limiting the opportunity for examining trends across clinics. Improving the measurement of mental health care should be a priority for health care systems seeking to expand reach of evidence-based psychotherapies. This will require not only mandates but also resources to build efficient databases with common data elements and ensure that the databases are consistently populated with accurate and useful data.

We find it interesting that medium reach teams shared similarities with both high and low reach teams. They were more similar to high reach teams with regard to team mission, team engagement and infrastructure outside the team. However, they resembled both high and low reach teams with regard to clinic operations and staff perceptions. The medium reach teams we studied had adopted an evidence-based psychotherapy team mission more recently than the high reach teams and one of these medium reach teams embraced this mission alongside of a symptom management treatment model. The experience of medium reach teams suggests that the transition from low to higher reach requires first adopting an evidence-based psychotherapy team mission to which the team leader and staff are committed while ensuring infrastructure outside the team to meet the needs of patients not addressed through this focused mission. It may be that changes in specific clinic operations and staff perceptions of benefits to patients proceed more slowly or variably.

There are limitations to this work. While this was very large sample for qualitative research, only 10 PTSD teams at nine medical centers were studied. A quantitative study would be needed to test the relationship between the themes we identified and reach. Second, we compared and contrasted teams that varied in the proportion of therapy patients who received CPT or PE. We did not examine the number CPT or PE sessions received by patients and thus do not know whether high reach teams were more successful in keeping patients engaged in these evidence-based psychotherapies. The relationship between reach and dose is an important topic for future research. Third, we used phone rather than in person interviews at two of our nine sites. Although these two sites provided surveys and program documentation (e.g., patient brochures and manuals), the fact that we did not conduct face to face interviews and observe team member interactions may have limited the depth of our understanding of these two sites. Fourth, we examined factors affecting implementation of PE and CPT in specialized PTSD outpatient clinics, and the findings cannot be generalized to other clinic environments, including the general mental health clinics where clinicians treat a range of presenting problems in addition to PTSD. Last, we used terms familiar to mental health leaders and clinicians to describe the major themes. This was done to make our findings accessible to stakeholders and help bridge the research to practice gap. However, this decision may create a challenge for researchers seeking to integrate findings on implementation and sustainability across studies. We hope that we have created enough of a cross-walk from our findings to the Sustainability Model to mitigate this disadvantage.

Limitations notwithstanding, our findings contribute to the literature on contextual factors associated with sustained implementation of evidence-based trauma-focused psychotherapies and provide information that can guide future research and quality improvement efforts. Strategies to support the implementation and sustainability of evidence-based psychotherapies should foster the development and implementation of a clinic mission that prioritizes evidence-based psychotherapies. This will require developing and empowering leaders and staff to establish clinic infrastructure and operations and design comprehensive mental health services that facilitate, normalize and reinforce delivery of evidence-based psychotherapies while also negotiating responsibilities and workload across clinics in the broader practice environment.

## Electronic supplementary material

Below is the link to the electronic supplementary material.

Supplementary material 1 (DOCX 17 KB)
