# Supplementary material for: Context Matters: Team and Organizational Factors Associated with Reach of Evidence-Based Psychotherapies for PTSD in the Veterans Health Administration
Source: Adm Policy Ment Health. 2017 Jun 9;44(6):904–18. doi: 10.1007/s10488-017-0809-y (PMC5640758; doi:10.1007/s10488-017-0809-y)
Supplement: Supplementary file 1 — Supplementary material 1 (DOCX 17 KB) [file 10488_2017_809_MOESM1_ESM.docx]

**Data Supplement 1**. Profession of staff participants (*N* = 96) by site

|  | Site Label | | | | | | | | | |
| --- | --- | --- | --- | --- | --- | --- | --- | --- | --- | --- |
|  | 7 | 5^a^ | 2 | 4 | 3 | 9 | 6 | 1 | 8 | Regional staff^b^ |
| Total (*n*) | 10 | 11 | 7 | 15 | 13 | 8 | 12 | 8 | 9 | 3 |
| Profession |  |  |  |  |  |  |  |  |  |  |
| Clinical psychologist | 5 | 8 | 4 | 9 | 5 | 7 | 3 | 4 | 3 | 1 |
| Social Worker | 2 | 1 | 2 | 2 | 4 | 1 | 2 | 2 | 3 | 0 |
| Psychiatrist | 2 | 1 | 1 | 2 | 2 | 0 | 3 | 1 | 3 | 2 |
| Primary care physician | 0 | 0 | 0 | 0 | 0 | 0 | 1 | 0 | 0 | 0 |
| Clinical nurse specialist | 0 | 0 | 0 | 0 | 0 | 0 | 3 | 1 | 0 | 0 |
| Peer support specialist | 0 | 0 | 0 | 1 | 0 | 0 | 0 | 0 | 0 | 0 |
| Psychology or social work trainee | 1 | 1 | 0 | 1 | 2 | 0 | 0 | 0 | 0 | 0 |

^a^ This site had two PTSD teams but shared some staff

^b^Regional staff were affiliated with sites 3, 7, 8, 9
